# Evaluation of early interventional treatment opportunity of the elderly & high-risk patients with non-ST segment elevation acute myocardial infarction

**DOI:** 10.12669/pjms.315.7881

**Published:** 2015

**Authors:** Zhiqiang Liu, Lipei Zhao, Yibo Li, Zhifang Wang, Lingling Liu, Fucheng Zhang

**Affiliations:** 1 Zhiqiang Liu, Department of Cardiology, Xinxiang Central Hospital, Xinxiang 453000, China; 2 Lipei Zhao, Department of Cardiology, Xinxiang Central Hospital, Xinxiang 453000, China; 3 Yibo Li, Department of Cardiology, Xinxiang Central Hospital, Xinxiang 453000, China; 4 Zhifang Wang, Department of Cardiology, Xinxiang Central Hospital, Xinxiang 453000, China; 5 Lingling Liu, Department of Cardiology, Xinxiang Central Hospital, Xinxiang 453000, China; 6 Fucheng Zhang, Department of Cardiology, Xinxiang Central Hospital, Xinxiang 453000, China

**Keywords:** non-ST segment elevation acute myocardial infarction (NSTEMI), Early interventional treatment, High risk

## Abstract

**Objective:**

To investigate the effect of treatment on prognosis of patients with different timing of early interventional treatment for non-ST segment elevation acute myocardial infarction (NSTEMI).

**Methods:**

Forty two cases above 75 years old, diagnosed with high-risk on NSTEMI, were selected in cardiology department of Xinxiang central hospital. They were randomly divided into two groups: 22 in group A and 20 in group B. Group A was performed PCI surgery within 12 hours after the onset while group B from 12 to 24 hour after the onset. Major adverse cardiovascular events (including death, heart failure readmission rates after ischemia, malignant arrhythmias, again target vessel revascularization) and bleeding data were recorded at the three terms of hospitalization, one month after the onset and six months after the onset.

**Results:**

Angina, malignant arrhythmia and heart failure during hospitalization can be reduced after interventional treatment carried out within 12 hours after the onset. Readmission rates after ischemia, heart failure and the incidence of death can be significantly reduced after interventional treatment carried out during 1-6 month after the onset with no significant increase in bleeding rate.

**Conclusion:**

In the treatment of elderly patients with NSTEMI, early interventional treatment is safe and effective.

## INTRODUCTION

Non-ST segment elevation acute myocardial infarction (NSTEMI) is the major onset form of coronary artery disease, with high incidence, complications and poor prognosis, which is a serious threat to human health.^1^,^2^ About 10% of patients are die within six months and 20% of patients with myocardial infarction and re-onset need angioplasty.^3^ Non-ST segment elevation acute coronary syndrome (NSTE-ACS) in patients with multivessel coronary artery often has multiple severe stenosis, but not yet complete occlusion in the infarct-related vessels.^4^

This pathological characteristic determines that the acute treatment principles of NSTE-ACS and ST-segment elevation myocardial infarction (STEMI) are different.^5^,^6^ The near-term death risk in patients with NSTE-ACS is less than that in patients with STEMI, but serious risk of coronary events, such as the medium and long-term mortality, myocardial infarction, is not low.^7^ It is reported that revascularization can improve the medium and long-term prognosis of patients with NSTE-ACS. Elderly and high-risk NSTEMI patients with past medical histories including heart failure, myocardial infarction and revascularization are prone to serious arrhythmias or heart failure. Meanwhile, they have low left ventricular ejection fraction (LVEF), so early treatment on them is particularly significant. Early interventional treatment is important, but the choice on interventional timing remains controversial.

This study observes the incidences of adverse cardiovascular events at different timing of interventional treatment on patients with NSTEMI after the onset.

## METHODS

### Subjects

42 cases diagnosed with NSTEMI were selected in cardiology department of Xinxiang central hospital from March 2011 to March 2012. This study was conducted in accordance with the declaration of Helsinki with approval from the Ethics Committee of Xinxiang Central Hospital. Written informed consent was obtained from all participants.

### Inclusion criteria

(1) above 75 years old; (2) the diagnostic criteria for NSTEMI in accordance with ECS / ACCF / AHA / WHF diagnostic criteria for myocardial infarction in 2007, namely: having evidence of myocardial necrosis (high cTnT, cTnI and / or CK-MB), and the ECG without ST-segment elevation^3^; (3) GRACE score> 140.

### Exclusion criteria

(1) Less than 75 years old; (2) patients combined with autoimmune diseases, blood diseases and malignancy; (3)patients with body temperature> 38°C and / or combined with any infected system; (4) patients combined with liver and kidney dysfunction or severe respiratory diseases; (5) peripheral vascular diseases or peripheral vascular thrombosis diseases; (6) patients with recent trauma or surgery.

### Exclusion criteria for AMI

(1) non-myocardial infarction confirmed by coronary angiography; (3) AMI related with intervention or coronary artery bypass graft treatment; (4) AMI secondary to AMI caused by an imbalance of myocardial oxygen supply and consumption, such as, coronary thrombosis, coronary spasm, anemia and so on.

### Methods

42 cases which met the above criteria were randomly divided into two groups: 22 in group A and 20 in group B. Group A was performed PCI surgery within 12 hours after the onset while group B from 12 to 24 hour after the onset. The data for all selected cases was collected including gender, age, past medical history of smoking, drinking, hypertension, diabetes, dyslipidemia, myocardial infarction. Blood was collected for checking enzymes, troponin, blood routine test, blood lipids and detecting indicators of CK, CK-MB, CTNI, WBC, N, CHO, HDL, LDL and so on. All patients were given conventional medication (aspirin, clopidogrel bisulfate, statins, nitrates, ACEI or ARB). The two groups were run on coronary angiography at different time periods, preoperative 300mg chewable aspirin and clopidogrel bisulfate tablets 300mg. And the appropriate instruments and stents were selected for coronary stent implantation according to the nature of the disease. Successful criteria for PCI was the opening of occluded vessels (TIMI 2-3 blood level), no residual stenosis, no dissection and no serious complications. Major adverse cardiovascular events (including death, heart failure readmission rates after ischemia, malignant arrhythmias, again target vessel revascularization) and bleeding data were recorded at the three terms of hospitalization, one month after the onset and six months after the onset.

### Statistical analysis

The data in this study was analyzed by SPSS 17.0 (SPSS Inc, Chicago, IL, USA). Measurement data was performed using *t* test and expressed as X±S. Count data was tested by x^2^. P<0.05 was considered statistically significant.

## RESULTS

### General data

The general information, such as age, sex, smoking, drinking, hypertension, diabetes mellitus, myocardial infarction, PCI, heart failure, was compared between A, B groups and it was not statistically significant (P> 0.05). Table-I

**Table-I:** Comparison of general data between group A and B.

General data	Group A	Group B	P value
Case	22	20	
Age	80.41±3.11	80.30±3.28	0.912
Gender (Male / Female)	11/11	13/7	0.366
Smoking history	12	13	0.377
Drinking history	9	13	0.121
Hypertension history	16	14	1.00
Diabetes history	11	11	0.767
MI history	8	8	1.00
HF history	11	8	0.551

### The results of coronary angiography in both groups

Forty two cases had coronary angiography and PCI. Table-II, Eight cases was single vessel lesion, accounting for 19%, 18 were double vessel lesions, accounting for 42.9%, 17 were three vessel lesions, accounting for 38.1%. 4 cases were anterior descending artery lesion, two were right coronary artery lesion, two were circumflex artery lesion; six cases were both anterior descending artery lesion and circumflex artery, eight were anterior descending artery and right coronary artery lesion, 4 were circumflex and right coronary artery lesion. Stent implantation was performed after balloon dilatation in all cases, successfully, no death and interventional complications.

**Table-II:** The results of coronary angiography in both groups.

Group	Single vessel lesion	Double vessel lesions	Three vessel lesions
Group A	5	10	7
Group B	3	8	9
P value	0.700	0.764	0.527

### Comparison of cardiovascular events in the two groups of patients hospitalized

Compared with group A, recurrent angina rate, malignant arrhythmia rate and heart failure rate were high in group B and there is a significant difference (P <0.05).Table-III

**Table-III:** Comparison of cardiovascular events in the two groups of patients hospitalized.

Group	Recurrent angina	Recurrent MI	Malignant arrhythmias	Heart failure	Death
Group A	2	0	2	1	0
Group B	8	3	8	6	0
P value	0.03	0.099	0.030	0.040	

### Comparison of cardiovascular events and bleeding in the two groups of patients in a period of one month after onset

Compared with group A, ischemic rehospitalization rate, heart failure rate and mortality were high and there is a significant difference (P <0.05). Table-IV

**Table-IV:** Comparison of cardiovascular events and bleeding in the two groups of patients in a period of one month after onset.

Group	Ischemic rehospitalization rate	PCI	Malignant arrhythmias	Heart failure	Bleeding	Death
Group A	0	0	0	0	0	0
Group B	5	2	3	4	0	1
P value	0.018	0.221	0.099	0.043	0	0.476

### Comparison of cardiovascular events and bleeding in the two groups of patients in a period of 6 months after onset

Compared with group A, ischemic rehospitalization rate, heart failure rate and mortality were high and there is a significant difference (P <0.05). Table-V

**Table-V:** Comparison of cardiovascular events and bleeding in the two groups of patients in a period of 6 months after onset.

Group	Ischemic rehospitalization rate	PCI	Malignant arrhythmias	Heart failure	Bleeding	Death
Group A	3	1	2	3	0	0
Group B	10	2	6	9	0	5
P value	0.016	0.435	0.111	0.035	0.366	0.02

## DISCUSSION

Non-ST segment elevation acute myocardial infarction (NSTEMI) is coronary heart disease in emergency, with high incidence, complications and poor prognosis, which is a serious threat to human health. NSTEMI patients are different from ST segment elevation acute myocardial infarction in pathological features. Considerable studies have shown that rich lipid, thin fibrous cap and a large number of inflammatory cell for infiltration in NSTEMI patients could make plaque rupture; moreover the release of a large number of active substance led to platelet adhesion and aggregation, which finally formed thrombosis. The thrombus is white, and its treatment is also different from ST segment elevation acute myocardial infarction, which is still controversial. Some scholars believed patients with early NSTEMI undergoing PCI increased mortality and bleeding rate. However, some studies showed that the shorter the period from admission to intervention, the lower the mortality and the incidence of nonfatal myocardial infarction within 30 days, and there was no relevance between serious bleeding events and interventional treatment timing. In recent years, domestic and foreign research institutions have done many studies on the choice of therapeutic methods for patients with NSTEMI, and most studies mainly focus on the selection of thrombolytic or interventional therapy.^8^

Elderly and high-risk NSTEMI patients with past medical histories including heart failure, myocardial infarction and revascularization are prone to serious arrhythmias or heart failure.^9^ Meanwhile, they have low left ventricular ejection fraction (LVEF). Therefore, early intervention in the treatment on elderly patients with NSTEMI is significant important and is recognized.^10^,^11^ However, the choice on interventional timing remains controversial. In FRISC II interventional treatment the median time is 4 days, RITA3 is three days, TACTICS-TIMI18 is 25 hours.^12^,^13^

Montalescot et al.^14^ reported that patients with NSTEMI treated by intervention within 24-48 hours had lower mortality and the incidence of nonfatal myocardial infarction than patient treated within 24 hours and after 48 hours. Tricoci et al.^15^ found that the shorter the period from admission to intervention, the lower the mortality and the incidence of nonfatal myocardial infarction within 30 days, and there was no relevance between serious bleeding events and interventional treatment timing.

This study enrolled elderly NSTEMI and high-risk patients with GRACE score> 140 treated by intervention method within 12 hours or within 12-24 hours after onset, and all patients underwent PCI successfully. The results showed that angina, malignant arrhythmia and the incidence of heart failure reduced after intervention within 12 hours; ischemic rehospitalization rate, heart failure and the incidence of death reduced after intervention in 1-6 months; there was no significant increase in bleeding rate. Meanwhile, quality of patient life improved significantly. Therefore, early interventional method is safe and effective in the treatment on elderly patients with NSTEMI.
